# Developmental pyrethroid exposure and age influence phenotypes in a *Chd8* haploinsufficient autism mouse model

**DOI:** 10.1038/s41598-022-09533-x

**Published:** 2022-04-01

**Authors:** Jessica A. Jiménez, Jeremy M. Simon, Wenxin Hu, Sheryl S. Moy, Kathryn M. Harper, Chih-Wei Liu, Kun Lu, Mark J. Zylka

**Affiliations:** 1 Curriculum in Toxicology and Environmental Medicine, The University of North Carolina at Chapel Hill, Chapel Hill, NC 27599 USA; 2 UNC Neuroscience Center, The University of North Carolina at Chapel Hill, Chapel Hill, NC 27599 USA; 3 Carolina Institute for Developmental Disabilities, The University of North Carolina at Chapel Hill, Chapel Hill, NC 27599 USA; 4 Department of Psychiatry, The University of North Carolina at Chapel Hill, Chapel Hill, NC 27599 USA; 5 Department of Genetics, The University of North Carolina at Chapel Hill, Chapel Hill, NC 27599 USA; 6 Department of Environmental Sciences and Engineering, The University of North Carolina at Chapel Hill, Chapel Hill, NC 27599 USA; 7 Department of Cell Biology and Physiology, The University of North Carolina at Chapel Hill, Chapel Hill, NC 27599 USA

**Keywords:** Disease model, Autism spectrum disorders

## Abstract

Hundreds of genes have been associated with autism spectrum disorder (ASD), including loss-of-function mutations in chromodomain helicase DNA binding protein 8 (*Chd8*). Environmental factors also are implicated in autism risk and have the potential to exacerbate phenotypes in genetically sensitized backgrounds. Here we investigate transcriptional and behavioral phenotypes in a *Chd8* haploinsufficient (*Chd8*^V986*/+^) mouse line exposed to the pesticide deltamethrin (DM) from conception to postnatal day 22. Vehicle-exposed *Chd8*^V986*/+^ mice displayed ASD-associated phenotypes, including anxiety-like behavior and altered sociability, replicating a previous study with this mouse line. A core set of genes was altered in *Chd8*^V986*/+^ mice at multiple ages, including *Usp11*, *Wars2*, *Crlf2*, and *Eglf6,* and proximity ligation data indicated direct binding of CHD8 to the 5’ region of these genes. Moreover, oligodendrocyte and neurodegenerative transcriptional phenotypes were apparent in 12 and 18 month old *Chd8*^V986*/+^ mice. Following DM exposure, the mutant mice displayed an exacerbated phenotype in the elevated plus maze, and genes associated with vascular endothelial cells were downregulated in the cerebral cortex of older *Chd8*^V986*/+^ animals. Our study reveals a gene x environment interaction with a *Chd8* haploinsufficient mouse line and points to the importance of investigating phenotypes in ASD animal models across the lifespan.

## Introduction

Autism spectrum disorder (ASD) is characterized by deficits in social communication, restricted interests, and repetitive behaviors^1^. ASD is a public health concern, affecting ~ 1.5% of the population in developed countries^2^. Our understanding of the genetics underlying ASD has greatly advanced in recent years, with the identification of common variants and hundreds of rare de novo gene mutations associated with the disorder^3–5^. Genetic factors contribute ~ 60%-90% to ASD risk based on heritability studies^6–8^, with the remaining risk unaccounted for, a portion of which may be attributed to environmental factors.

Environmental risk factors for ASD include prenatal maternal infection, valproic acid use during pregnancy, and exposure to air pollution during pregnancy^9^. Prenatal exposure to pesticides, including maternal proximity to pesticide applications, has also been reproducibly associated with increased autism risk^6,9–11^. Moreover, environmental factors are hypothesized to affect individuals with specific genetic backgrounds rendering them more susceptible to harmful environmental influences. Environmental risks for ASD have the potential to go largely undetected in large epidemiological studies that sample a large genetically heterogenous pool, with insignificant power to detect modest effects in a specific genetic subpopulation. Thus, greater efforts are needed to understand gene-environment (GxE) interactions that contribute to ASD risk. For example, the extent to which pesticide exposures influence phenotypes in ASD model mice has been largely unexplored.

The majority of ASD susceptibility genes converge on key molecular pathways, including transcription/chromatin remodeling and synaptic signaling/neurotransmission^4^. Chromodomain helicase DNA binding protein 8 (*CHD8*) is one of the most commonly mutated genes in people with ASD and may function to modulate the transcription of many genes^12,13^. We recently developed a haploinsufficient mouse model with an ASD-linked loss-of-function mutation in *Chd8* (*Chd8*^V986*/+^) that displays modest ASD-associated phenotypes, including anxiety-like behavior and macrocephaly^14^, in agreement with other *Chd8* mutant mouse models^15–20^. Moreover, we found that many of the behavioral phenotypes became more prominent with age^14^.

Mutations in genes associated with synaptic signaling/neurotransmission, including mutations in *SCN2A*, the gene encoding the sodium channel Nav1.2, have also been identified in patients with intellectual disability and ASD^21^. Sodium channel inhibition is a common mechanism by which insecticides function to lethally alter neuronal activity in pests^22^, which may explain the epidemiological correlation between pesticide exposure and ASD. Pyrethroids are one such class of insecticides that have been epidemiologically associated with ASD and attention deficit hyperactivity disorder (ADHD) risk^10,23^. The association between pyrethroid exposure and intellectual disabilities is of concern as 89% of the homes sampled in a nationwide study had detectable pyrethroid levels in their dust samples^24^.

Genetic and environmental perturbations during development are predicted to have the greatest potential to contribute to ASD risk^5^. In a recent mouse study, early prenatal and postnatal exposure to the pyrethroid, deltamethrin (DM), below the developmental no observed adverse effect level (NOAEL), was found to induce ADHD-like symptoms in young mice^25^, but ASD-related phenotypes were not examined. Considering the association between pyrethroid exposure and ASD, and the strong genetic component of the disorder, environmental risk factors may augment ASD genetic predisposition and increase disease risk. Here we investigate ASD-like transcriptional and behavioral phenotypes in a *Chd8* haploinsufficient mouse model when exposed to DM at prenatal and early postnatal ages.

## Methods

### Mice and perinatal exposure

All data presented in this study are from mice obtained by breeding *Chd8*^V986*/+^ male mice^14^ with C57BL/6J wild-type (WT) females. This breeding scheme was used because pup survival was reduced when raised by *Chd8*^V986*/+^ females^14^. Upon confirmation of a vaginal plug, embryonic day 0 (E0), females were fed the first dose of oil control or DM (3 mg/kg) (ChemService, N-11579) mixed into peanut butter (Skippy, Hormel Foods), and were then fed DM or oil in peanut butter every 3 days up until weaning at postnatal day (PND) 22, to replicate work by Richardson et al.^25^. To rigorously control for litter effects^26^, only one animal of each sex and genotype were used per litter. Mice were raised in a facility with a 12:12-light:dark cycle with ad libitum access to food (Teklad 2020X, Envigo, Huntingdon, UK) and water. Genomic DNA was isolated from tail clips by incubating samples at 95 °C in 50 μL of 25 mM NaOH/0.2 mM EDTA for 30 minutes (min), followed by the addition of 100 μL of 40 mM Tris HCl (pH 5.5). Genotyping was performed by polymerase chain reaction (PCR) amplification of genomic DNA with primers: (F) 5′ GCTAAGACAGAAATCTGATCTA TTACCAGTAGA and (R) 5′ GGTCTTGAGATCCC CAAAATCCTTAA followed by MboI restriction enzyme digestion (BioLabs, R0147L), to distinguish WT (227 bp product) and mutant (149 bp and 78 bp products) alleles. Animal protocols in this study were approved by the Institutional Animal Care and Use Committee at the University of North Carolina at Chapel Hill and were performed in accordance with these guidelines and regulations. All studies are reported in accordance with ARRIVE (Animal Research: Reporting of In Vivo Experiments) guidelines.

### Behavior assessments

Subjects used in behavioral tests were male mice exposed to vehicle control or DM from shortly after conception (E0) to PND 22. Mice were evaluated at 6 months of age in the first round of behavioral tests and 12 months of age for the second round and included 10 vehicle-exposed *Chd8*^V986*/+^, 10 vehicle-exposed WT, 12 DM-exposed *Chd8*^V986*/+^, and 10 DM-exposed WT mice. Tests were conducted in this order (age in weeks): first round, elevated plus maze (26 weeks), open-field (27 weeks), and social approach in a three-chamber choice task (28 weeks); and second round, elevated plus maze (52 weeks), open-field (53 weeks), and social approach in a three-chamber choice task (54 weeks). An additional cohort of mice exposed to vehicle (6 *Chd8*^V986*/+^ and 7 WT) or DM (6 *Chd8*^V986*/+^ and 7 WT) was tested in the open-field at 6 weeks of age to evaluate anxiety-like phenotypes in younger animals. All behavioral testing methods have been previously described^14^.

### Elevated plus maze

Mice were given one 5 min trial on the plus maze, which had two walled arms (the closed arms, 20 cm in height) and two open arms. The maze was elevated 50 cm from the floor, and the arms were 30 cm long. Mice were placed on the center section (8 cm × 8 cm) and allowed to freely explore the maze. Measures were taken of time on and the number of entries into the open and closed arms.

### Open-field test

Exploratory activity in a novel environment was assessed by a 1–1.5 h trial in an open-field chamber (41 cm × 41 cm × 30 cm) crossed by a grid of photobeams (VersaMax system, AccuScan Instruments). At 6 months, mice were tested for 1 h and at 12 months mice were tested for 1.5 h to determine persistence of changes in activity and anxiety-like behavior. Counts were taken of the number of photobeams broken during the trial in 5 min intervals, with measures taken of locomotion (total distance traveled), rearing movements, and time spent in the center region of the open-field, an index of anxiety-like behavior. A separate cohort of 6 week old mice was tested by a 1.5 h trial in an open-field chamber (45 cm × 45 cm × 40 cm). The total distance moved by each mouse in the open arena was recorded by camera (Sony) connected to the EthoVision software (Noldus Wageningen).

### Three-chamber choice task

Mice were evaluated for social preference in a three-chamber choice task^27^. The social testing apparatus was a rectangular, three-chambered box fabricated from clear Plexiglas. Dividing walls had doorways allowing access into each chamber. An automated image tracking system (Noldus Ethovision) provided measures of time spent in 5 cm proximity to each cage and numbers of entries into each side of the social test box. The procedure consisted of three 10 min phases: a habituation period, a test for sociability, and a test for social novelty preference. During habituation, the mouse was allowed to explore the chamber for 10 min. For the sociability assay, mice were given a choice between being in the proximity of an unfamiliar conspecific (stranger 1) versus an empty cage. The C57BL/6J adult male (stranger 1) mouse was enclosed in a small Plexiglas cage drilled with holes. An identical empty Plexiglas cage was placed in the opposite side of the chamber. The test mouse was allowed to explore the entire social test box for a 10 min session. In the social novelty phase, mice were given a choice between the already-investigated stranger 1 versus a new unfamiliar C57BL/6J male mouse (stranger 2). The test mouse was given an additional 10 min to explore the social test box.

### Histology

Tissue used for histology was from a cohort of dams exposed to vehicle control (n = 8) or DM (n = 9) from E0 to E15. Embryonic brains from one animal/sex/genotype/litter were dissected in phosphate-buffered saline (PBS) and fixed overnight in 4% paraformaldehyde in 0.1 M phosphate buffer, pH 7.4 at 4 °C, followed by cryoprotection in 30% sucrose in 0.1 M phosphate buffer, pH 7.4 at 4 °C. Tissue used for histology to assess vasculature and oligodendrocytes was from 12 month old male mice exposed to vehicle control or DM from E0 to PND 22. Mice were perfused with 4% paraformaldehyde in 0.1 M phosphate buffer, pH 7.4. The brains were dissected and immersion fixed in the same fixative buffer, freshly prepared, for 24 h, followed by cryoprotection in 30% sucrose in 0.1 M phosphate buffer, pH 7.4 at 4 °C. Using a cryostat, embryonic brains were cut into 30 μm sections and adult brains were cut into 40 μm sections for glial cell staining and 80 μm sections for vasculature staining. Sections were rinsed with PBS before blocking in 10% normal donkey serum (NDS; EMD-Millipore, S30-100ML) with 0.1% Triton (Sigma, T8787) in PBS for 30 min. Sections were incubated with primary antibodies diluted in 5% NDS with 0.1% Tween (BP337, Fisher Scientific) in PBS overnight at 4 °C. The following day, sections were once again rinsed in 0.1% Tween/PBS and blocked with 10% NDS/0.1%Tween/PBS before application of a secondary antibody cocktail diluted in 5%NDS/0.1% Tween/PBS. After incubation with secondary antibodies for 2 h, sections were rinsed in PBS and incubated with DAPI (ThermoFisher, 62248) diluted in PBS (1:1500) for 5 min. Sections were rinsed again and floated onto SuperFrost Plus slides (Fisher Scientific, 12-550-15), briefly dried, and coverslipped with Fluoro Gel mounting medium (Electron Microscopy Sciences, 17985-10). The following primary antibodies were used at a concentration of 1:400: rat anti-EOMES (TBR2) (Invitrogen 14-4875-82), rabbit anti-TBR1 (Abcam, ab31940), and rabbit anti-Ki-67 (D3B5) (Cell Signaling, 9129). Rabbit anti-OLIG-2 (Millipore Sigma, AB9610) was used at 1:2000. Secondary antibodies (donkey anti-rabbit Alexa Fluor 568, A-10042, donkey anti-rat Alexa Fluor 488, A-21208, and donkey anti-rabbit Alexa Fluor 647, A-31573) were purchased from Invitrogen and used at 1:1000. Images were acquired with a Zeiss LSM 710 confocal microscope using a 40× objective. Cell counts were quantified using Fiji (imageJ).

To label cells in S phase of the cell cycle, 5-ethynyl-2’-deoxyuridine (EdU) dissolved in saline was administered to pregnant dams at E15 by intraperitoneal (i.p.) injection at 50 mg/kg, 30 min before sacrificing. Following immunohistochemistry, brain sections were re-blocked in 10% NDS/0.1% Tween/1% 0.1 M glycine/PBS for 1 h. Sections were then treated with an EdU reaction solution (1.6 mM Alexa 488-azide, 4 mM CuSO4, 100 mM sodium ascorbate, 10 mM Tris pH 8.5 in PBS) for 1 h at room temperature before being washed with PBS and mounted. Images were acquired with a Zeiss LSM 710 confocal microscope using a 40X objective and cells were quantified using Fiji.

For vasculature staining, after blocking in 10% NDS/0.1% Triton/PBS for 30 min, Isolectin GS-IB_4_ (Alexa Fluor 488 Conjugate, Invitrogen I21411), diluted in 5% NDS/0.1% Tween/PBS at a concentration of 1:100, was added and incubated overnight at 4 °C. The following day, sections were rinsed in PBS and incubated with DAPI (ThermoFisher, 62248) diluted in PBS (1:1500) for 5 min. Sections were rinsed again and floated onto SuperFrost Plus slides (Fisher Scientific, 12-550-15), briefly dried, and coverslipped with Fluoro Gel mounting medium (Electron Microscopy Sciences, 17985-10). Z stack images were acquired with a Zeiss LSM 710 confocal microscope using a 20× objective. Z stack images were flattened to a maximum intensity projection and quantified in Fiji using the Vessel Analysis Plugin.

### Sample preparation and liquid chromatography/mass spectrometry analysis

Brain, placenta, and serum samples were extracted as describe previously^28^. In brief, 10 μL of 10 ng/mL DM-(phenoxy-D5) (Sigma) was added to the samples and incubated at room temperature for 30 min. The samples were extracted with 3 mL of acetone:hexane (2:8), followed by vortexing for 10 min. After centrifugation at 4000 rpm for 5 min, the organic extracts were collected. This step was repeated twice. The organic extracts were then combined, evaporated to dryness, and then reconstituted in 1 mL hexane. The extraction was loaded to Sep-pak silica solid phase extraction column (Waters; SKU: 186004615). The column was pre-washed with 5 mL hexane, and eluted with 5 mL hexane:ethyl acetate (90:10). The elution was collected, evaporated to dryness, and then dissolved in 50 μL of methanol.

Analysis of DM was performed using a Vanquish UHPLC system coupled with Q Exactive Hybrid Quadrupole-Orbitrap mass spectrometer (Thermo Fisher Scientific). LC separation of target analytes was achieved by a ACQUITY UPLC HSS T3 column (2.1 mm × 100 mm, 1.8 μm particle, Waters) using an isocratic elution with 200 μL/min of 99% buffer B (buffer A: 90/10 water/methanol with 5 mM ammonium acetate; buffer B: 10/90 water/methanol with 5 mM ammonium acetate). The total LC–MS/MS time was 7 min, column temperature was set to 40 °C, and sample injection volume was 10 μL. DM and its internal standard were detected by parallel reaction monitoring for the following target precursors: DM (C_22_H_19_NO_3_Br_2_): [M + NH_4_]^+^, *m*/*z* 521.0070; internal standard DM-D5 (C_22_H_14_D_5_NO_3_Br_2_): [M + NH_4_]^+^, *m*/*z* 526.0384. The higher-energy collisional dissociation (HCD) normalized collision energy was set as 15 with isolation window of 2.0 *m/z* and AGC target of 1e6 with maximum injection time of 250 ms. The Orbitrap resolution for full scan and MS2 scan was set to 70,000 and 35,000, respectively. The HESI source parameters used were the default settings optimized for 200 μL/min flow rate. The limit of quantification for DM was 0.5 ng/mL.

### Reverse transcription-quantitative PCR (RT-qPCR)

To quantify mRNA levels, total RNA was extracted from one cortical hemisphere of PND 5 WT and *Chd8*^V986*/+^ mice exposed to vehicle control or DM from E0 to PND 5, and from unexposed WT and *Chd8*^V986*/+^ 6 week old mice. Tissue was lysed in 1 mL of TRIzol (ThermoFisher, 15596026) using a tissue homogenizer, and RNA was extracted by following the Invitrogen TRIzol Reagent RNA extraction protocol. cDNA was synthesized with the Invitrogen SuperScript IV VILO Master Mix cDNA synthesis protocol (ThermoFisher, 11756050). PowerUp SYBR Green Master Mix (ThermoFisher, A25742) and the QuantStudio 5 Real-Time System (ThermoFisher) were used for the RT-qPCR. The following primers were used. *Wars2*_F: GCCCAGCACTTGGGATGT^29^; *Wars2*_R: GCAGCCAGCTCACCAATG^29^; *Crlf_F*: AGAGACTGGGAAAGGGAGAA; *Crlf_R*: CTGTCATTTGGTTGCACATTGA; *Egfl6_F*: GGCAGGGCACAAGAAGAATA; *Egfl6_R*: GCCGGTAATCAAAGAGCAAAC; *Usp11_F*: CCACGCATACAAGTGTTGCACC; *Usp11_R*: CTCAATCCGACCAGTCACCTCA; *Xrcc4*_F: GACTCGCAAACCACGGTATTA; *Xrcc4*_R: TACCTCTCAGTACTCCACTTCC; *Smug1*_F: TCCTCTCCACACTTCCTCTT; *Smug1*_R: CTGCCTCTGAGTTATGGGATTAC; *Fancc*_F: CTGTGGCTCTTGGTGTTCTAT; *Fancc*_R: GCATCAGGAGACGGTTGATTA; *Rpa2*_F: CAGCTTGGTGGAGTCTGCTT; *Rpa2*_R: TAGTCACAATCTGCGGTGGC; *Apc*_F: GTGGACTGTGAGATGTATGGGC; *Apc*_R: CACAAGTGCTCTCATGCAGCCT; *Olig2*_F: ATGCACGACCTCAACATCGCCA; *Olig2*_R: ACCAGTCGCTTCATCTCCTCCA; *Pdgfrα*_F: GGCCAGAGACATCATGCACGATTC; *Pdgfrα*_R: TCAGCGTGGTGTAGAGGTTGTCGAA; *Kdr*_F: CGAGACCATTGAAGTGACTTGCC; *Kdr*_R: TTCCTCACCCTGCGGATAGTCA; *Ptprb*_F: ATCCTCGTCCTGACCATCAGTG; *Ptprb*_R: GCTCCAGGTTACCATCAGCCTA; *Cnp*_F: CTCTACTTTGGCTGGTTCCTGAC; *Cnp*_R: GCTTCTCCTTGGGTTCATCTCC; *Mag*_F: CAGTTGCCAAGAGCCTGTACCT; *Mag*_R: TTCACTGTGGGCTTCCAAGGTG; *Mbp*_F: CCCAAGGCACAGAGACACGGG; and *Mbp*_R: TACCTTGCCAGAGCCCCGCTT. *Gapdh* was used for normalization: *Gapdh*_F: ATGACCACAGTCCATGCCAT and *Gapdh*_R: GCTTCACCACCTTCTTGATG.

### RNA extraction for sequencing

WT and *Chd8*^V986*/+^ mice exposed to vehicle control or DM from E0 to PND 5 were sacrificed at PND 5 and mice exposed to vehicle control or DM from E0 to PND 22 were sacrificed at 12 months of age by decapitation. A separate cohort of unexposed WT and *Chd8*^V986*/+^ mice were sacrificed at 18 months of age by decapitation. The brains were removed, and the cerebral cortex (one hemisphere) was dissected and stored at − 80 °C until RNA extraction. Samples were processed in 4 batches, including meticulous planning to ensure that equal numbers of each genotype and time point were centrifuged together, to minimize batch effects associated with any part of processing, handling, RNA isolation, or RNA-sequencing library preparation. To lyse the samples, 350 μl of Qiagen RLT Plus buffer (with 20 μL DTT per mL buffer) per 20 mg tissue was added. A hand-held motorized homogenizer was used to immediately homogenize the samples. The lysate (350 μL) was processed through a RNeasy Plus Mini Kit for RNA extraction following the manufacturer protocol (Qiagen, 74136). RNA concentration was measured using Qubit RNA Broad-Range Assay and stored at − 80 °C (Thermo Fisher Scientific, Q10211).

### RNA sequencing and pathway analysis

PolyA RNA-stranded libraries were prepared (TruSeq mRNA stranded, Illumina) and sequencing was performed at the New York Genome Center (NYGC). Sequencing was conducted on an Illumina NovaSeq with stranded paired-end 100 bp reads. Reads were filtered for a quality score of 20 or more in at least 90% of all bases using fastq_quality_filter in the FASTX toolkit 0.0.14 (http://hannonlab.cshl.edu/fastx_toolkit/index.html). Sequencing adapters were trimmed using cutadapt 1.12^30^, and reads were then aligned to the mm10 reference genome using STAR 2.5.2b^31^. All samples sequenced had a median depth (filtered reads that aligned to the genome) of 28 × 10^6^ read pairs. Transcripts were quantified using Salmon 0.11.3^32^, and differential expression was detected using DESeq2 1.26^33^, using a model that corrected for batch and/or sex/litter effects and a threshold of adjusted p-value < 0.05.

To determine which pathways were associated with differentially expressed genes (DEGs), we used gprofiler 0.1.6^34^. A unique pathway database was created consisting of MSigDB C2, C5, and HALLMARK classifications, as well as neurodevelopmental and neuropsychiatric disease gene sets that we curated from the literature^35^, and markers of cortical cell types from single-cell RNA-sequencing data^36^. Pathways were considered significant at adjusted p-value < 0.05. The variance-stabilizing transformation (VST)-normalized expression values, corrected for batch using limma 3.42.2^37^, for each genotype underlying these fold-changes, were then aggregated across all significant genes in each pathway by taking the average on a replicate by replicate basis. All fold-changes reported by DESeq2 were shrunken using the ‘ashr’ method 2.2–47^38^.

### Statistical analysis

For all experiments using PND 5 and 6 week old mice, and for histological analysis of embryonic brains, only one animal/sex/genotype was randomly selected from each litter. For all experiments using 6, 12, and 18 month old mice, only one male of each genotype was randomly selected from each litter. This approach was done to avoid litter bias effects^26^. To appropriately account for the litter effect in embryonic body and organ weights, these data were analyzed using a nonlinear mixed model approach by applying the R-package “nlme” to the data, with litter variable as a random effect. For each experiment, measures were taken by an observer blind to mouse genotype and prenatal exposure. Data were analyzed using two-way or repeated measures Analysis of Variance (ANOVA), with the factors genotype and exposure. Separate analyses were then carried out for the oil vehicle and DM groups, to determine genotype effects within each exposure condition. Fisher's protected least-significant difference (PLSD) tests were used for comparing group means only when a significant F value was determined. Within-group ANOVAs were used to determine side preference in a 3-chamber test. For all comparisons, significance was set at p < 0.05. Data presented in figures are means (± SEM).

## Results

### DM enters the developing fetus and is detected in the fetal brain following prenatal exposure

**Figure 1:**
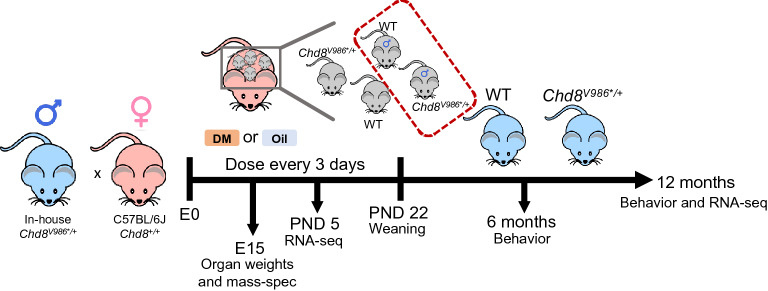
Experimental design. WT C57BL/6J females were bred with *Chd8*^V986*/+^ males. The dam was administered 3 mg/kg of DM or oil mixed into peanut butter every 3 days, beginning at E0. At E15, organs were weighed and DM was measured with mass spectrometry. RNA-sequencing was performed at PND 5 and 12 months. Male mice were assessed in behavioral experiments at 6 months and re-assessed at 12 months. Only one animal/sex/genotype was used from each litter. Males and females were assessed at E15 and PND 5.

To assess the effect of developmental DM exposure on autism-related phenotypes in WT and *Chd8*^V986*/+^ mutant animals later in life, WT females were bred with *Chd8*^V986*/+^ males. Upon confirmation of a vaginal plug (E0), females were fed the first dose of oil (vehicle control) or DM (3 mg/kg) mixed into peanut butter as a low stress means of chemical administration that more accurately represents exposure to pesticides via food in the human population^25^. This dose was selected by Richardson et al. to represent an exposure below the NOAEL^39^. The dam was fed DM or oil every 3 days up until embryonic day 15 (E15) (Fig. 1). At E15, DM was detected in the dams serum and brains via mass spectrometry (Fig. S1a). The dams body and brain weights were not altered (Fig. S1b,c). DM was also detected in the brain and placenta of E15 mice, confirming that DM enters the developing embryo (Fig. S1a). No DM was detected in any of the oil control samples. To assess the effects of DM on cell proliferation and maturation, confocal analysis was performed on E15 coronal brain sections spanning the ventricular/subventricular zones out to the cortical plate of the cerebral cortex. Although studies suggest that DM exposure may affect cellular proliferation^40,41^, no differences in neuronal proliferation, neuronal maturation, or brain weights were detected in E15 mice exposed to DM compared to vehicle-exposed animals (Figure S1e,g-l). Independent of the exposure, body and placenta weights were significantly heavier in oil- and DM-exposed *Chd8*^V986*/+^ embryonic mice compared to WT mice (Fig. S1d,f).

### Behavioral phenotypes in WT and *Chd8*^V986*/+^ mice following pre and early postnatal DM exposure

**Figure 2:**
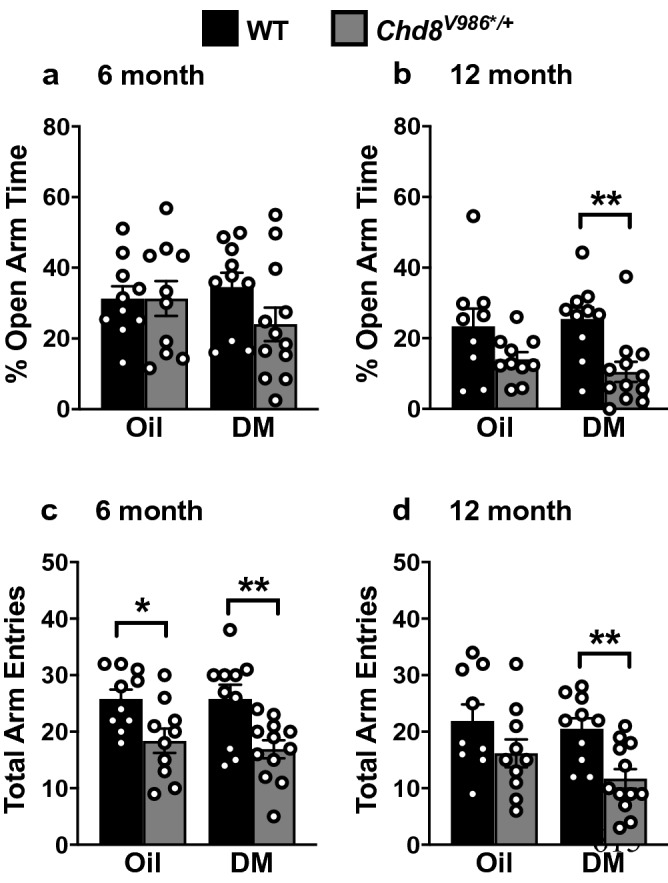
Increased anxiety-like behavior in *Chd8*^V986*/+^ mice following DM exposure. **(a,b)** Time spent in the open arms and **(c,d)** number of total arm entries in the elevated plus maze were assessed. Data are means (± SEM) for a 5 min test in an elevated plus maze. N = 10–12 mice per group. Fisher’s PLSD tests were used for comparing group means only when a significant F value was determined by using a two-way ANOVA, with genotype and treatment as factors. *p < 0.05; **p < 0.01.

**Figure 3:**
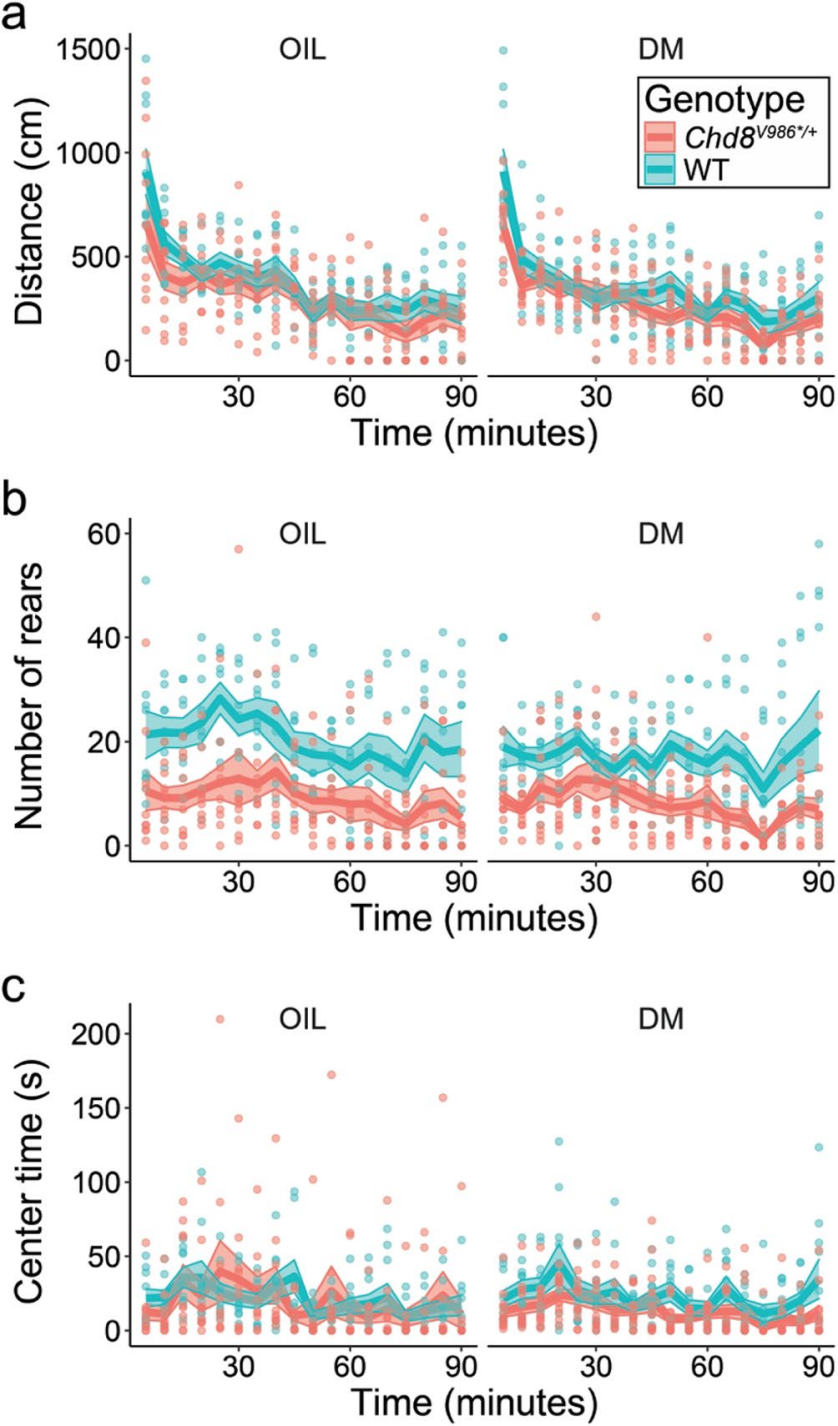
Open-field behavior in 12 month WT and *Chd8*^V986*/+^ mice following DM exposure or oil control. **(a)** Distance traveled, **(b)** number of rears, and **(c)** time in center. Cohorts were tested for 90 min at 12 months of age to determine persistence of changes in activity and anxiety-like behavior. Data are means (± SEM). N = 10–12 mice per group. Fisher’s PLSD tests were used for comparing group means only when a significant F value was determined by using a two-way ANOVA, with genotype and treatment as factors.

**Figure 4:**
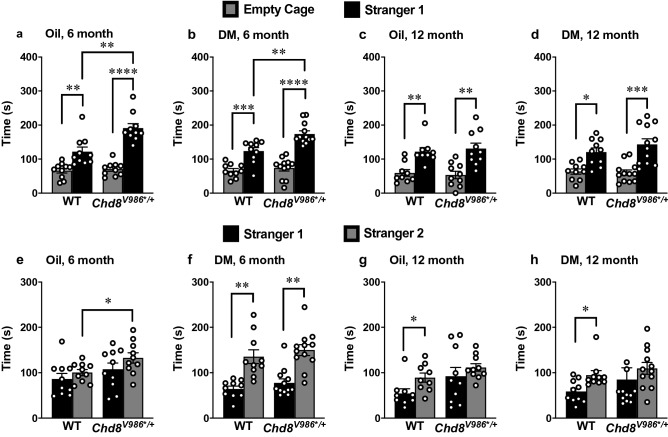
Social test in as a function of age and DM exposure in *Chd8*^V986*/+^ mice. Mice were tested at 6 and 12 months of age. **(a–d)** Sociability assessed by the time spent in 5 cm proximity to a caged stranger or an empty cage. **(e–h)** Social novelty preference tested by the time spent in 5 cm proximity to each caged stranger mouse. Bars represent mean ± S.E.M. for a 10 min test. N = 10–12 mice per group. Fisher’s PLSD tests were used for comparing group means only when a significant F value was determined by using a two-way repeated measures ANOVA for each exposure and age group. *p < 0.05, **p < 0.01, ***p < 0.001, ****p < 0.0001.

For behavioral tests, all subjects were male mice exposed to vehicle control or DM from shortly after conception (E0) to PND 22. Only one male of each genotype was used from each litter for behavioral and RNA-sequencing analysis, to avoid confounding our results with the litter effect (Fig. 1). *Chd8*^V986*/+^ mice demonstrated several behavioral phenotypes, including increased anxiety-like behavior in an elevated plus maze (Fig. 2), decreased rearing movements in the open-field (Fig. 3), and hyper-sociability in a three-chamber test (Fig. 4).

### Elevated plus maze

At 6 months of age, WT and *Chd8*^V986*/+^ mice spent similar percent of time in the open arms of the maze (Fig. 2a). In contrast, a significant main effect of genotype emerged at 12 months [F^1,37^ = 12.8, p = 0.001]. Post-hoc comparisons indicated that, in the DM exposure groups, *Chd8*^V986*/+^ mice had lower percent open arm time, in comparison to WT (Fig. 2b). This genotype difference did not reach significance in the vehicle-exposed groups (p = 0.0932).

Total arm entries were used to measure general activity during the test (Fig. 2c,d). Significant main effects of genotype were found for total entries at 6 months [F(1,38) = 16.89, p = 0.0002] and 12 months [F(1,37) = 10.47, p = 0.0026]. In both exposure groups, 6 month old *Chd8*^V986*/+^ mice had decreased numbers of total entries. In the 12 month old animals, these deficits in exploration were only found in the DM-exposed *Chd8*^V986*/+^ mice. Thus, differences in plus maze behavior between WT and mutant animals were most prominent in the *Chd8*^V986*/+^ mice exposed to DM.

### Open-field

In the open-field test, a significant genotype × time interaction for distance traveled was identified at 6 months [F(11,418) = 2.38, p = 0.0073] (Fig. S2a) and a significant genotype main effect was present at 12 months [F(1,629) = 5.44, p = 0.0252] (Fig. 3a). Separate analyses for each exposure condition revealed that significant genotype differences were only present in the DM exposure groups [genotype x time interaction; 6 month, F(11,220) = 2.5, p = 0.0057; 12 month, F(17,340) = 1.79, p = 0.028]. In both tests, the DM-exposed *Chd8*^V986*/+^ mice had significantly reduced activity in the first 5 min of the test (p < 0.05) (Fig. 3a, Figure S2a), in line with the exploratory deficits observed in the elevated plus maze (Fig. 2). A previous study found that prenatal DM exposure increased distance traveled in 6 week old mice, only when the last 60 min of a 90 min open-field trial were observed^25^. This phenotype was not observed at 6 weeks, 6 months or 12 months of age in the present study (Fig. S3).

Upright rearing is another form of exploratory activity. The *Chd8*^V986*/+^ mice exposed to either oil or DM had decreased rearing, in comparison to WT, at both testing ages [main effect of genotype; 6 month, F(1,38) = 24.44, p < 0.0001; 12 month, F(1,37) = 22.09, p < 0.0001] (Fig. 3b; Fig. S2b).

The overall analysis of time spent in the center region failed to indicate significant effects of either genotype or exposure (Fig. 3c; Fig. S2c). However, separate analyses for each exposure group revealed a significant main effect of genotype in the 12 month groups exposed to DM [F(1,20) = 4.87, p = 0.0391]. In these groups, the *Chd8*^V986*/+^ mice spent less time in the center region than WT, suggesting the prenatal exposure to DM led to increased anxiety in the open-field.

### Three-chamber task

In the three-chamber task, at both 6 and 12 months of age, all experimental groups had significant preference for spending more time in proximity to the cage containing the stranger mouse, versus the empty cage [within-group comparisons following overall repeated measures ANOVAs, significant effect of side, 6 month, F(1,38) = 136.69, p < 0.0001; 12 month, F(1,37) = 64.37, p < 0.0001] (Fig. 4a–d). Six month old *Chd8*^V986*/+^ mice spent significantly more time with the stranger mouse than WT mice did (Fig. 4a,b). This phenotype was not altered with DM exposure and was not apparent in 12 month old animals (Fig. 4c,d).

Our findings also indicate differential effects of prenatal exposure and genotype on social novelty preference, dependent on age. At 6 months, only the mice exposed to DM, but not oil, exhibited a shift in preference to the newly-introduced stranger 2 [exposure x side interaction, F(1,38) = 8.12, p = 0.007; effect of side, F(1,38) = 24.92, p < 0.0001] (Fig. 4e,f). Although a significant side preference was not observed in the oil-exposure groups, the mutant mice spent increased time in proximity to stranger 2, in comparison to WT [main effect of genotype, F(1,38) = 9.84, p = 0.0033]. At 12 months, only the WT groups demonstrated significant social novelty preference [within-group comparisons following overall repeated measures ANOVAs, significant effect of side, F(1,37) = 5.0, p = 0.0315], while the *Chd8*^V986*/+^ mice failed to discriminate between the first stranger mouse and the newly-introduced stranger 2 [main effect of genotype, F(1,37) = 8.37, p = 0.0064] (Fig. 4g,h).

### DM exposure is associated with reduced expression of genes involved in cellular adhesion and vasculature development in *Chd8*^V986*/+^ mice

**Figure 5:**
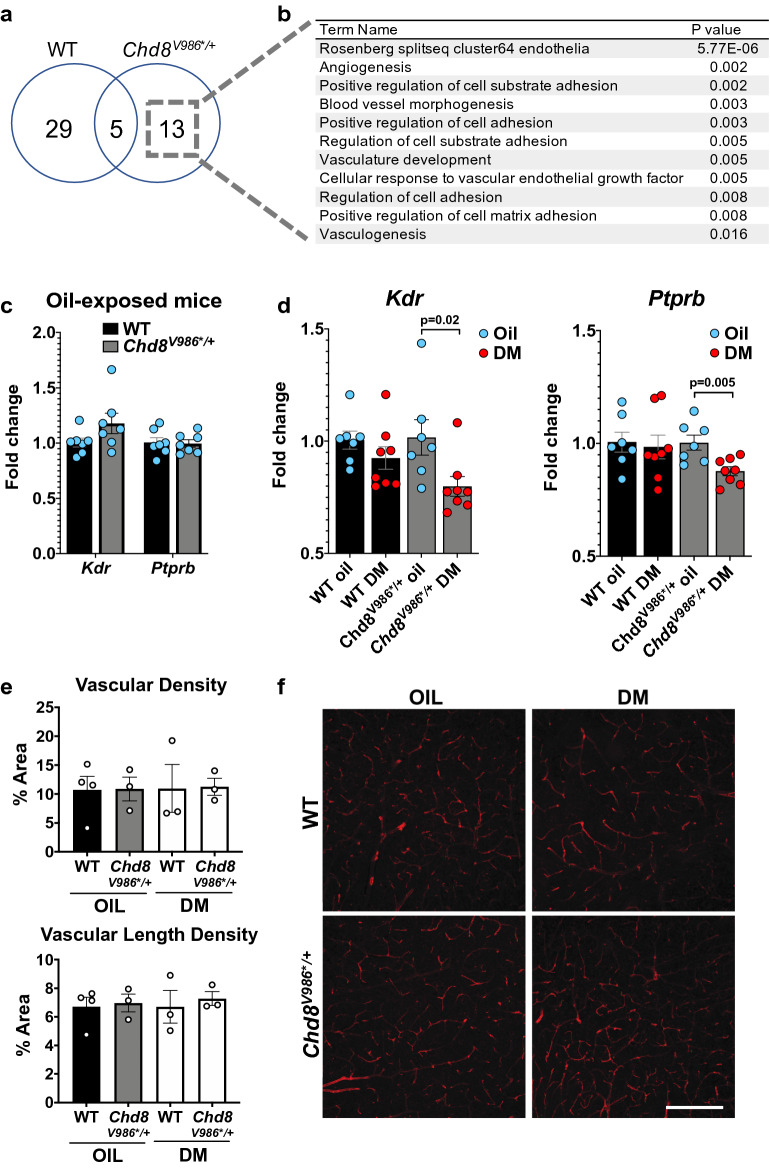
DM exposure is associated with altered cellular adhesion and vasculature development in *Chd8*^V986*/+^ mice**.** **(a)** Number of DEGs associated with DM exposure identified with RNA-sequencing of cortical tissue from 12 month old mice pre- and postnatally exposed to DM. N = 10–12 mice per group. **(b)** DM-induced DEGs from *Chd8*^V986*/+^ mice converge on downregulated pathways involved in cellular adhesion and vascular growth. **(c)** Cortical expression of vascular endothelial cell-related genes from RT-qPCR analysis of 12 month old WT and *Chd8*^V986*/+^ mice exposed to oil. **(d)** Effect of DM exposure on cortical gene expression *Kdr* and *Ptprb* genes from RT-qPCR analysis of 12 month WT and *Chd8*^V986*/+^ mice. N = 7–8/genotype/treatment. **(e,f)** Vascular density and vascular length density assessed with isolectin B4 staining and quantified. Each symbol represents the average of two 1024 × 1024 pixel images obtained from different cortical tissue sections for one brain (n = 3–4). Scale bar represents 200 μm. Data represent means ± SEM.

We performed bulk RNA sequencing of cortical tissue from oil- and DM-exposed 12 month old WT and *Chd8*^V986*/+^ mice. DM exposure altered 29 and 13 genes in WT and *Chd8*^V986*/+^ mice, respectively, relative to WT and *Chd8*^V986*/+^ oil controls (Fig. 5a, Supplementary data file 1). Interestingly, many of these genes converge on pathways involved in cell adhesion and angiogenesis. Pathway analyses revealed a downregulation of these pathways in *Chd8*^V986*/+^ animals exposed to DM (Fig. 5b, Supplementary data file 3). We performed RT-qPCR to validate that the downregulation of genes associated with vascular endothelial cells, *Kdr* and *Ptprb*, was unique to the 12 month old *Chd8*^V986*/+^ animals exposed to DM (Fig. 5d). Immunohistochemistry staining, however, revealed no difference in vascular length or vascular density in DM-exposed mice (Fig. 5f,g).

### Differentially expressed genes in *Chd8*^V986*/+^ mice suggest altered DNA repair pathways and a link to neurodegenerative phenotypes

**Figure 6:**
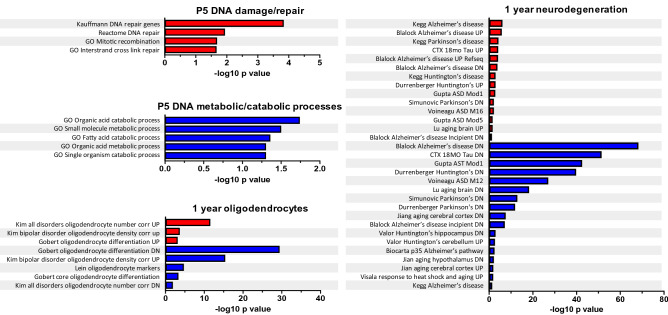
Summary of select upregulated and downregulated pathways in oil-exposed *Chd8*^V986*/+^ mice**.** Data derived from bulk RNA-sequencing analysis of cortical tissue from PND 5 (P5) and 1 year old *Chd8*^V986*/+^ mice compared to WT control. Bar graphs represent the significance of pathways in four categories.

Previously, we reported that pathways associated with synaptic and neuronal projections and sodium channel activity were reduced in embryonic *Chd8*^V986*/+^ mice, but equalized to WT levels postnatally. At 12 months of age, *Chd8*^V986*/+^ mutant animals displayed a reduction of genes associated with endoplasmic reticulum stress, chaperone-mediated protein folding, and the unfolded protein response^14^. We validated and extended these sequencing efforts in the current study by performing bulk RNA sequencing of cortical tissue from oil-exposed PND 5 and 12 month old WT and *Chd8*^V986*/+^ mice. We detected 229 DEGs in PND 5 *Chd8*^V986*/+^ mice compared to WT control mice (Supplementary data file 1). These genes converge on pathways that suggest a downregulation of catabolic and metabolic processes and an upregulation of DNA repair (Fig. 6, Fig. S5c, Supplementary data file 3).

From 12 month old oil-exposed samples, we identified 2,435 DEGs in *Chd8*^V986*/+^ mice compared to WT animals (Supplementary data file 1), a substantially greater number of DEGs than were previously identified in 12 month *Chd8*^V986*/+^ mice^14^, which may be attributed to an increased sample size (10 animals per genotype in the present study as opposed to 6 in our previous study). In agreement with our previous study, genes associated with protein phosphorylation, protein folding, and the unfolded protein response were reduced in 12 month *Chd8*^V986*/+^ mice (Supplementary data file 3). Using our curated pathway database (see “Methods”), we also identified an association of these DEGs with genes linked to neurodegenerative diseases, including Alzheimer’s disease, Parkinson’s disease, and Huntington’s disease in 12 month *Chd8*^V986*/+^ mice (Fig. 6, Supplementary data file 3). Further, oligodendrocyte-related pathways were dysregulated in 12 month old *Chd8*^V986*/+^ mice (Fig. 6, Supplementary data file 3). Specifically, our data showed an increase in expression of the mature oligodendrocyte marker, *Apc,* but decreased expression of myelin-related genes, *Cnp*, *Mag*, and *Pdgfra* (Supplementary data file 1). We validated these findings using RT-qPCR (Fig. S4). Further, expression of myelin basic protein (*Mbp*) was not altered in *Chd8*^V986*/+^ cortical samples (Fig. S4a, Supplementary data file 1). Differences in total oligodendrocytes were not evident via changes in *Olig2* gene expression or immunohistochemistry staining, which suggests an altered balance between mature and immature oligodendrocytes in 12 month *Chd8*^V986*/+^ mice (Fig. S4b,c, Supplementary data file 1).

### Temporal regulation of DEGs in *Chd8*^V986*/+^ mice identifies a set of genes tightly regulated by CHD8

**Figure 7:**
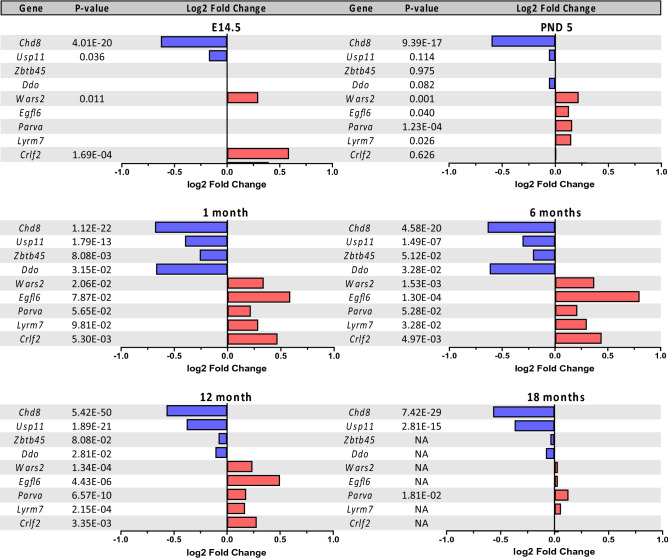
Core set of DEGs in oil-exposed *Chd8*^V986*/+^ mice relative to WT mice, determined by bulk RNA-sequencing of cortical tissue at E14.5, PND 5, 1 month, 6 months, 12 months, and 18 months. These core genes were significantly changed at multiple ages. Adjusted p-values and log2 fold changes from E14.5, 1 month, and 6 month old animals were obtained from our previous study^14^.

Our published sequencing data from *Chd8*^V986*/+^ animals at E14.5, 1 month, 6 months, and 12 months of age were compared with a new sequencing data set consisting of PND 5, 12 month and 18 month old animals (Supplementary data file 2). This led to the identification of a set of consistently up/down-regulated genes in the mutant animals at multiple ages (Fig. 7). Five of these “core” DEGs with the greatest significance/magnitude of change in *Chd8*^V986*/+^ mice, include *Chd8*, *Usp11*, *Wars2*, *Crlf2*, and *Eglf6,* and were validated in a different cohort of 6 week old animals (Fig. S5a). In a recent study, targeted DamID in utero was used to characterize CHD8 binding in developing embryonic mouse cortex^42^. Intriguingly, data derived from this study suggests that CHD8 directly associates with *Usp11*, *Wars2*, *Crlf2*, and *Eglf6* near the promoter or proximal to the first exon (Fig. S5b).

## Discussion

### DM reaches the developing fetus following prenatal exposure

We found that the pyrethroid pesticide DM, administered orally to pregnant dams at an environmentally-relevant level, enters the fetal brain, indicating that the developing fetus can be directly exposed to DM. DM has been reported to reduce neuronal proliferation, maturation and differentiation, and to increase apoptosis in fetuses exposed to 1.2 mg/kg/day (dam body weight) from E10.5 to E15.5^40^. DM did not induce changes to corticogenesis in the current study, possibly because we treated pregnant females with 3 mg/kg DM every 3 days, whereas Guo et al. treated at a slightly lower dose (1.2 mg/kg) but on a daily basis. Our dosing strategy of 3 mg/kg DM every 3 days has been shown to reduce hippocampal neurogenesis in adult mice^41^, but the amount of DM that enters the fetal brain is much lower than that of the dam (Fig. S1), suggesting that higher developmental exposure doses may be required to alter fetal neurogenesis.

### *Chd8* haploinsufficiency reproducibly affects specific behaviors in mice

*Chd8*^V986*/+^ mice show anxiety-like phenotypes and reduced exploration in the open-field, in agreement with our recent study with a different cohort of animals (Fig. 3, Fig. S2)^14^. Increased anxiety and reduced exploratory behaviors were also newly observed in the elevated plus maze (Fig. 2). However, the overall lower exploratory activity of *Chd8*^V986*/+^ mice, along with a pronounced reduction in rears performed in the open field task, could suggest motor deficits, which limits the interpretation of a specific anxiety-related phenotype in the *Chd8*^V986*/+^ mice. In contrast with our previous study^14^, increased interest in social novelty was not observed in *Chd8*^V986*/+^ mice. However, *Chd8*^V986*/+^ mice displayed increased sociability, which is a novel behavioral observation in the *Chd8*^V986*/+^ mice, and validates a relationship between *Chd8* haploinsufficiency and increased social behaviors (Fig. 4)^14,16,18,19^. This behavior could also indicate a deficit in inhibitory behavioral control of aggressiveness or a risky social approach, which were not behaviors investigated in this study. Together, these data suggest subtle differences in sociability and anxiety-like phenotypes in *Chd8*^V986*/+^ that present themselves uniquely, dependent age and testing cohorts. We also cannot exclude the possibility that these subtle differences are due to testing animals in the same task at multiple ages.

Interestingly, behavioral differences between WT and *Chd8*^V986*/+^ mice were often more pronounced when comparing DM-exposed animals. DM led to a more significant reduction in elevated plus maze behavior in *Chd8*^V986*/+^ mice, and was associated with reduced activity in the open-field (Figs. 2b,d and 3a). Further, DM-exposed mice of both genotypes displayed an increased interest in social novelty at 6 months (Fig. 4f). These results suggest that prenatal and early postnatal DM exposure may affect behavior in adult mice, exacerbating existing behavioral deficits. However, no consistent effect of treatment, indicated by a two-way ANOVA, was observed across behavioral endpoints, which warrants a limited interpretation of the data.

Studies on the neurobehavioral toxicity associated with DM exposure in developing animals were recently reviewed^43^. Comparisons across DM-induced developmental toxicity studies are challenging due to differences in study design, exposure periods, dose, and species tested. Increased locomotor activity in mice following developmental DM exposure has been reported in 6 week^25^ and 4 month old mice^44^. While we modeled our study to precisely replicate Richardson et al. by using the same dosing strategy and minimizing the influence of the litter effect, we did not observe an increase in locomotor activity at 6 weeks, 6 months, or 12 months of age (Fig. 3, Figure S2, Figure S3). These differing results may stem from differences in testing environments, animal handling, and/or sample size (n = 4–7^25^ and 10–12 in the current study). However, the effect of DM on locomotor behavior in rodent models is inconsistent in the current literature. A number of studies report that rats exposed to DM during gestation were less active than controls when tested later in life^45–47^.

Developmental DM exposure in rodents has also been associated with increased impulsivity and reduced attention^25^, deficits in working memory in a Y-maze^25,48^, and impaired learning and memory in the Morris water maze task^47,49^. Anxiety-like behavior has also been reported in acutely exposed adult rats showing reduced arm entries, reduced time in open arms, and fewer center crossings in the elevated plus maze^50^. Together, these studies suggest that developmental DM exposure may alter exploratory behavior and cognition in offspring, manifesting as changes in locomotor activity, impulsivity, increased anxiety, and impairments in learning and memory, dependent on the dose administered and study design.

### DM exposure is associated with altered cellular adhesion and vasculature development

Gene expression changes in 12 month old *Chd8*^V986*/+^ mice following prenatal and early postnatal DM exposure were linked with perturbed cell to cell adhesion properties and vascular development (Fig. 5, Supplementary data file 3), although the biological significance of these differences is unclear, as we found no change in vascular length or vascular density (Fig. 5).

A dose dependent increase in damage to angiogenesis in zebrafish larvae exposed to DM has been reported^51^. This damage was associated with a decrease in expression of vascular endothelial cell genes (*fli-1* and *flk1*) indicating that DM may inhibit angiogenesis by downregulating expression of these genes^52^. RNA-sequencing data from zebrafish embryos treated with DM from fertilization to 48 h postfertilization highlighted that the most significantly altered biological GO term pathways included cell–cell adhesion and cell–cell communication^53^, in agreement with our current study of 12 month old animals exposed to DM prenatally and early postnatally. In rodent pups, repeated exposure of the pyrethroid, cypermethrin, led to an increased blood brain barrier permeability that normalized 43 days after withdrawal^54^. Together with our study, these data suggest that chronic DM exposure may alter cellular adhesion properties and vascular growth in developing organisms.

### Transcriptional signatures in *Chd8*^V986*/+^ mice

Twelve month old *Chd8*^V986*/+^ mice displayed a reduction of genes associated with endoplasmic reticulum stress, chaperone-mediated protein folding, and the unfolded protein response, reproducing our previous study^14^. In order to gain more insight into the implications of transcriptional differences between WT and *Chd8*^V986*/+^ mice, a pathway database was created consisting of MSigDB C2, C5, and HALLMARK classifications, as well as neurodevelopmental and neuropsychiatric disease gene sets^35^, and markers of cortical cell types from single-cell RNA-sequencing data^36^. Interestingly, 12 and 18 month old *Chd8*^V986*/+^ mice displayed transcriptional signatures associated with neurodegenerative diseases (Fig. 6, Supplementary data file 3).

Neurodegeneration has been suggested to play a role in the loss of neurological function in children with ASD who have experienced regression and loss of previously acquired skills^55^. Neurodevelopmental and neurodegenerative disorders present surprising commonalities in their pathological mechanisms, including early signs of synaptic degeneration and perturbed metabolic function^56^. Neuroinflammation, which has been reported to characterize many neurodegenerative diseases including Parkinson’s disease, Alzheimer’s disease, and multiple sclerosis, is also associated with ASD and implicates a role of inflammation in ASD etiology^57–59^. Further, ASD may develop as a consequence of neurodegenerative processes as the frequency of ASD-linked symptoms correlates with the progressing severity of cognitive impairments in individuals with dementia^60^. In addition, increased frequency of parkinsonism has been identified among ASD individuals older than 39 years^61^.

CHD8 has also been shown to promote oligodendrocyte lineage development and myelination^62^. Oligodendrocyte-related pathways were dysregulated in 12 and 18 month old *Chd8*^V986*/+^ mice (Fig. 6, Supplementary data file 3). Gene expression analysis suggests increased mature oligodendrocytes and a reduction in myelin-related genes *Cnp*, *Mag*, and *Pdgfra* (Fig. S4, Supplementary data file 1). However, expression of *Mbp* did not differ between animals (Fig. S4). Reduced expression of these myelin-related genes were also reported in oligodendrocytes of *Chd8* haploinsufficient mice in vitro^63^, and decreased expression of oligodendrocyte-specific genes have been reported in the brains of other *Chd8* haploinsufficient mice and humans with ASD^63^. The specific reduction of CHD8 in oligodendrocytes was associated with abnormal behavioral phenotypes in mice, similar to the behavioral phenotypes reported in this study, including a reduced rearing response, increased social interactions, and increased anxiety-like behaviors^63^. Thus, directing research efforts toward older ASD animal models with neuroinflammatory phenotypes that are not present early in life, may shed light on ASD etiology that has been overlooked.

By influencing chromatin compaction and accessibility, CHD8 partakes in fundamental biological processes, including transcription, cellular proliferation, and DNA damage repair^64^. These functions may be reflected in the DEGs from PND 5 *Chd8*^V986*/+^ mice that suggest an altered DNA repair pathway in the mutant animals (Fig. 6, Fig. S5c, Supplementary data file 3). Interestingly, *CHD8* is mutated in breast cancer, gastric cancers, colorectal cancers, and prostate cancer^65–67^, diseases associated with aberrant cell proliferation. The possible parallels between cancer and increased fetal body and placental weight (Fig. S1) and postnatal brain weight^14^ are intriguing and necessitate further study. Further, CHD8 functions as a negative regulator of the tumor suppressor protein p53. While the full *Chd8*^*-/-*^ knockout is embryonically lethal, deletion of p53 has been shown to rescue some of this developmental arrest^68^. Recently, CHD8 has been associated with lengthening of the G1 phase as neural progenitors transition from a proliferative to a differentiated state^69^. Thus, faster progression of *Chd8*^V986*/+^ neural progenitors through G1 and the G1/S checkpoint, may result in an increased demand on DNA repair pathways.

### CHD8 regulates a core set of genes

In the current study, we identify a set of genes that are up/downregulated in *Chd8*^V986*/+^ mice at different ages (Fig. 7). CHD8 likely regulates expression of these genes by associating directly with their promoters and/or gene bodies (Fig. S5b)^42^, although identifying genuine CHD8 targets has proved challenging^70,71^. Rather than acting as a DNA sequence-specific transcription factor, several mechanisms underlying CHD8 binding specificity have been suggested, including the recognition of specific chromatin signatures generated by methylated lysine residues on free histone tails, and recruitment via protein–protein interactions^71,72^. How CHD8 functions to upregulate some genes and downregulate other genes remains to be elucidated. These conserved CHD8 target genes could be used to better understand the mechanisms by which CHD8 regulates gene expression in the developing and adult brain.

## Conclusions

Our study addresses the susceptibility of WT and *Chd8*^V986*/+^ mice to DM-induced developmental neurotoxicity. We replicated the increased anxiety-like behavior identified in *Chd8*^V986*/+^ mice. While we noted an exacerbated phenotype in the mutant mice following DM exposure, an effect of treatment on behavior was not obvious. Further, our work investigating the transcriptional profile of *Chd8*^V986*/+^ mice across their lifespan highlights genes that are tightly associated with *Chd8* expression and identifies a neurodegenerative transcriptional phenotype in 12 and 18 month *Chd8*^V986*/+^ mice. Overall, this study sheds light on the role of CHD8 throughout development and adulthood and underscores the need to assess ASD animal models at older ages.

## Supplementary Information

Supplementary Information 1.

Supplementary Information 2.

Supplementary Information 3.

Supplementary Information 4.

## Data Availability

Raw and processed RNA-sequencing data generated during this study have been deposited to the Gene Expression Omnibus (GEO) under accession GSE190466.
