# Supplementary material for: Developmental pyrethroid exposure and age influence phenotypes in a Chd8 haploinsufficient autism mouse model
Source: Sci Rep. 2022 Apr 1;12:5555. doi: 10.1038/s41598-022-09533-x (PMC8975859; doi:10.1038/s41598-022-09533-x)
Supplement: Supplementary file 1 — Supplementary Information 1. [file 41598_2022_9533_MOESM1_ESM.pdf]

Developmental pyrethroid exposure and age influence phenotypes in a *Chd8* haploinsufficient autism mouse model

Jiménez et al.

Supplemental Information

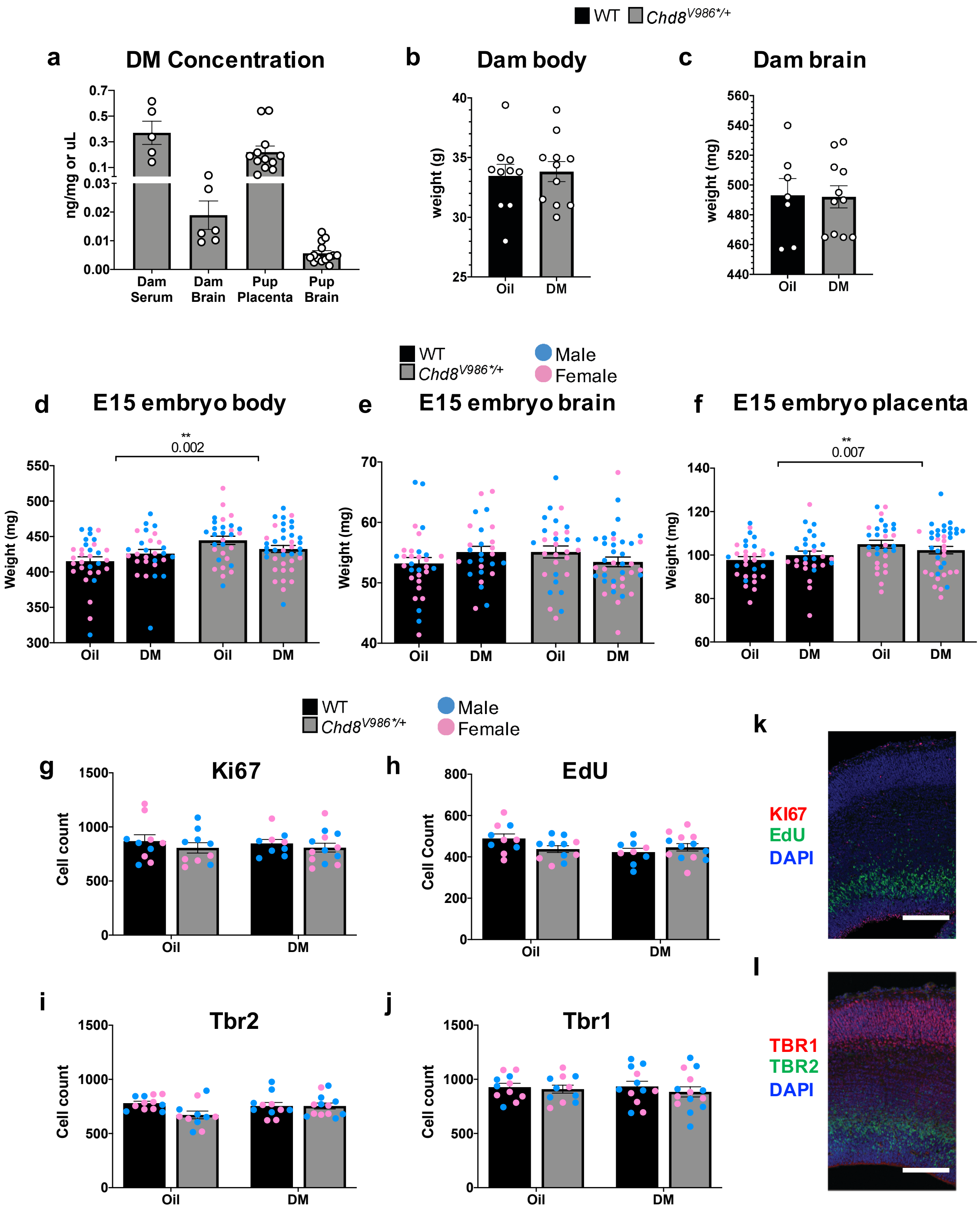

Figure S1. DM enters the developing fetal brain but altered corticogenesis was not observed. (a) DM is detected in the dam and fetal tissue at E15, (b-f) but does not affect the weight of the dam, fetus, or fetal tissue. (g,h) No changes in neuronal proliferation (Ki67 and EdU) or (i,j) neuronal maturation (Tbr2 and Tbr1) in the cortex and ventricular zone of the fetal brain at E15 were observed. Total cell counts are graphed. Each symbol represents the average of the total cell counts from at least two 2000 X 3000 pixel images obtained from different sections for one brain (n = 9 – 13). Data represents means ± SEM. (k,l) Representative immunohistochemistry images of the fetal cortex and ventricular zone are shown. Scale bars represent 150 μm.

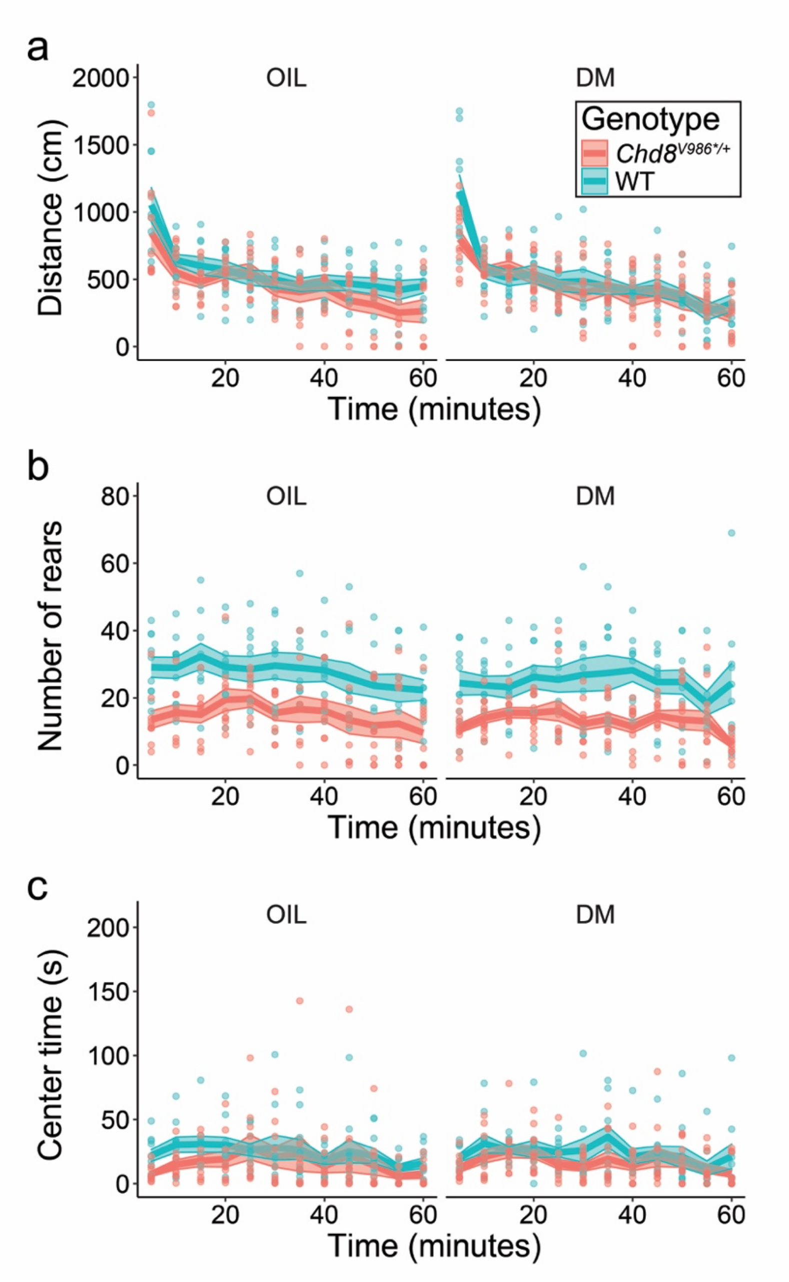

Figure S2. Open-field behavior in 6 month WT and *Chd8*$^{V986*/+}$ mice following DM exposure or oil control. (a) Distance traveled, (b) number of rears, and (c) time in center. Cohorts were tested for 60 min at 6 months of age. N = 10-12 mice per group. Fisher's PLSD tests were used for comparing group means only when a significant F value was determined by using a two-way ANOVA, with genotype and treatment as factors.

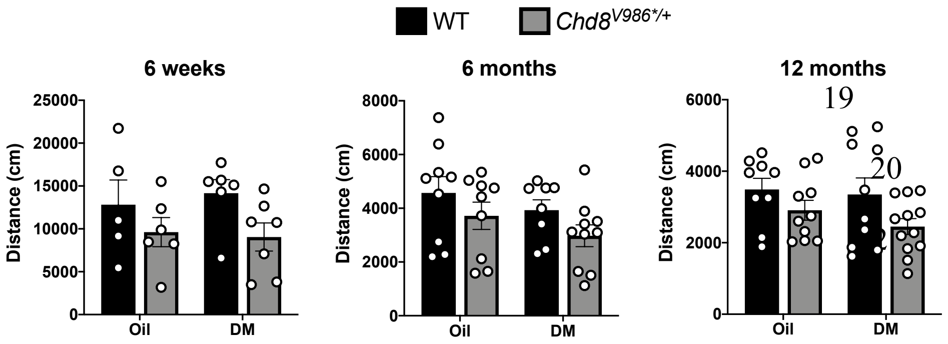

Figure S3. Open-field behavior in mice assessed at 6 months and then re-assessed at 12 months. A separate group of animals was tested at 6 weeks. Two-way ANOVA identified a significant effect of genotype at 6 weeks (p=0.0432) and 12 months (p=0.0292). Data represents means ± SEM.

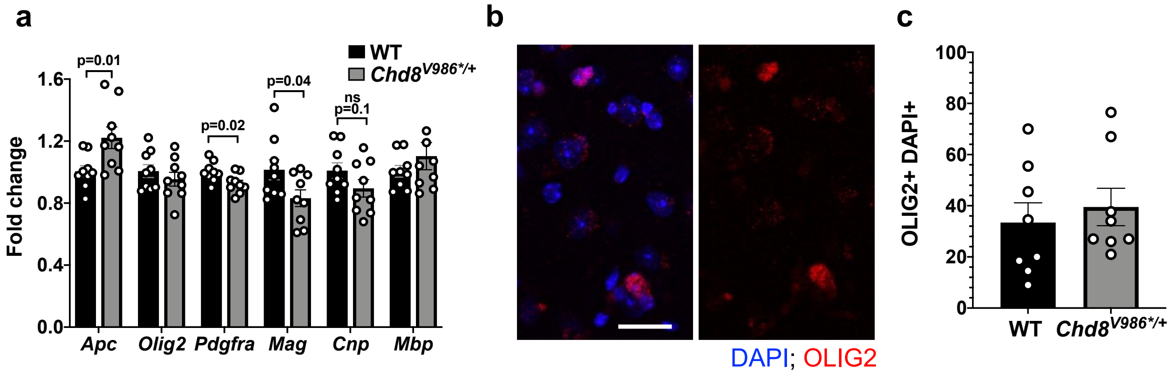

Figure S4. Oligodendrocyte signatures in *Chd8*$^{V986*/+}$ mice. (a) Cortical expression of oligodendrocyte marker genes from RT-qPCR analysis of 12 month WT and *Chd8*$^{V986*/+}$ mice (n = 9 / genotype). Data represents means ± SEM; ns = not significant. (b) Representative immunohistochemistry images of oligodendrocytes in cortical tissue sections from 12 month old *Chd8*$^{V986*/+}$ male brains. Scale bars represent 20 μm. (c) Total cell counts are graphed. Each symbol represents the average of the total cell counts from two 2800 x 4500 pixel images obtained from different sections for one brain (n = 8 / genotype). Data represents means ± SEM.

Figure S5. Core set of DEGs in *Chd8*$^{V986*/+}$ mice relative to WT mice. (a) Cortical gene expression from RT-qPCR analysis of 6 week old WT and *Chd8*$^{V986*/+}$ mice. (b) CHD8 binding at loci of interest obtained from Wade et al. and plotted using IGV 2.8.9 (1). CHD8-associated represents genomic interactions from CHD8 fused to an E. coli DNA adenine methyltransferase domain (Dam) or Dam only control, delivered by *in utero* electroporation to characterize CHD8 binding in developing embryonic mouse cortex. Representations of the mouse mm10 reference genome are shown below tracks in black. Height of the y-axis is scaled to show the peak for each track separately. (c) Cortical expression of DNA repair-related genes from RT-qPCR analysis of PND 5 WT and *Chd8*$^{V986*/+}$ mice. Data represents means ± SEM. W=WT, H=*Chd8*$^{V986*/+}$, M=male, F=female.

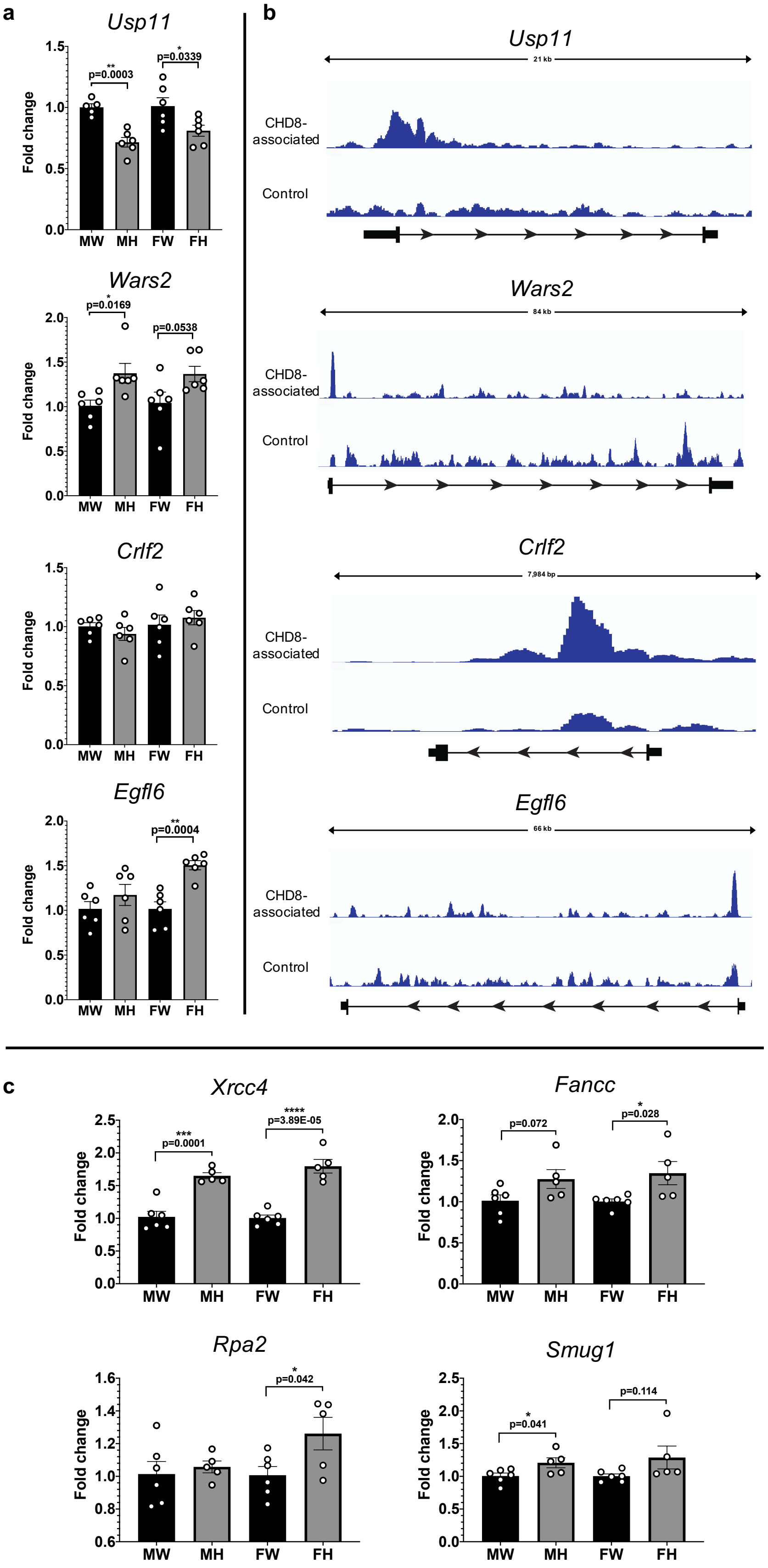

Supplementary data file 1. Normalized expression and differential expression (comparing genotypes and exposure conditions) results for all genes in PND 5 and 12 month cortical samples.

Supplementary data file 2. Normalized expression and differential expression (comparing genotypes) results for all genes in 18 month cortical samples.

Supplementary data file 3. Functional pathways that are significantly up or downregulated in PND 5, 12 month, and 18 month old cortical samples. Only comparisons with significant pathways enriched among them are listed.

References

1. Wade AA, van den Ameele J, Cheetham SW, Yakob R, Brand AH, Nord AS. In vivo targeted DamID identifies CHD8 genomic targets in fetal mouse brain. iScience. 2021;24(11):103234.
